# Editorial: Biosynthesis and regulation of plant specialized metabolisms

**DOI:** 10.3389/fpls.2023.1319666

**Published:** 2023-11-09

**Authors:** Weiwei Zhang, Feng Xu, Moonhyuk Kwon

**Affiliations:** ^1^ College of Horticulture and Gardening, Yangtze University, Jingzhou, China; ^2^ Division of Life Science, Anti-aging Bio Cell factory Regional Leading Research Center (ABC-RLRC), Plant Molecular Biology and Biotechnology Research Center (PMBBRC), Gyeongsang National University, Jinju, Republic of Korea

**Keywords:** specialized metabolites, flavonoids, terpenoids, alkaloids, biosynthetic pathway, regulatory factor

Plant-specialized metabolites such as flavonoids, polyketides, terpenoids, glucosinolates, and phenolic compounds, more commonly known as secondary metabolites are organic compounds produced via specialized metabolism. They are multifunctional metabolites that play an important role in the adaptation of plants to their environment, such as protection against natural enemies and pests, assistance in pollination, and protection against ultraviolet light damage. Based on the physiological activities, and pharmacological effects, plant-specialized metabolites have been widely used for the prevention and treatment of human diseases like tumors, aging, and cardiovascular disorders. The distribution of specialized metabolites in plants is unique to species, organs, tissues, and growth stages, resulting in complex and diverse biosynthetic pathways. The synthesis of plant-specialized metabolites is controlled by genetic material and regulated by a variety of biotic and abiotic factors in the environment.

During the growth and development of plants, they produce a large number of specialized metabolites that serve various cellular functions necessary for physiological processes. The synthesis and proper accumulation of these specialized metabolites are tightly controlled, and a complex regulatory network formed by enzyme genes and transcription factors (TFs) plays a key role in the metabolism of specialized metabolites. In this Research Topic, some of the articles provide some new insights into the regulation of the synthesis of different specialized metabolites. Wang et al. combined metabolomic and transcriptomic analyses to identify four structural genes involved in glucosinolate and soluble sugar biosynthesis (*BraA05gAOP1*, *BraA04gAOP4*, *BraA03gHT7*, and *BraA01gHT4*) and two transcription factors (*BraA01gCHR11* and *BraA07gSCL1*). Xu et al. discovered that *TcbZIP60* directly binds to the E-box/G-box motifs in the promoters of pyrethroid synthesizing genes *TcCHS* and *TcAOC* and activate their expression. Further studies revealed that transient overexpression of *TcbZIP60* increased the expression level of pyrethrin biosynthesis genes, resulting in a significant accumulation of pyrethrins. Conversely, silencing of *TcbZIP60* led to reduced pyrethrin accumulation and the expression of related genes. These results suggest that *TcbZIP60* is able to regulate the biosynthesis of pyrethrins. Xu et al. revealed the relationship between jasmonic acid (JA) and aspalathoside VI biosynthesis in *Dipsacus asperoides*. They discovered that triterpenoids, JA and TFs together regulate aspalathoside VI biosynthesis in *Dipsacus asperoides*.

Plants achieve functional flexibility under the influence of environmental factors through the production of specialized metabolites. A large number of studies have shown that the production of these specialized metabolites is associated with stress and defense response signals and that changes in external conditions will cause changes in specialized metabolites at the physiological and molecular levels. With the development of biological techniques, the application of metabolomics and transcriptomics technologies has greatly facilitated these studies. Specialized metabolites usually act as defense molecules to protect plants under various adverse conditions. In this Research Topic, Park et al. found that anthocyanins and two flavonoids were significantly increased, while all types of caffeoylquinic acids (CQAs) and flavonols were significantly decreased in *Ligularia fischeri* under drought stress; RNA sequencing led to the final identification of potential drought-responsive genes including up-regulated flavonoid synthetase (*LfFNS*) and anthocyanidin-O-glucosyltransferase (*LfA5GT1*), which are important for flavonoids and anthocyanins production by *Ligularia fischeri* in response to drought stress. In another study, Lim et al. treated mungbean (*Vigna radiata*) sprouts with salt stress and found that the content of several phenylpropanoid metabolites including catechin, chlorogenic acid, p-coumaric acid, and ferulic acid was significantly increased in mungbean sprouts under mild salt stress conditions, and at the same time, several key enzyme genes and transcription factors involved in the biosynthesis of phenylpropanoid and flavonoid compounds were significantly upregulated. The composition of light can influence the metabolism of various specialized metabolites. Lim et al. evaluated the changes of metabolites in soybean seedlings under different light conditions, finding that the duration of light exposure regulates the content of kaempferol glycoside, the predominant flavonol in soybeans, with longer light exposure resulting in higher levels. Most isoflavones increased in response to red and blue lights, but daidzein increased only in response to red light. Blue light was observed to stimulate the accumulation of kaempferol-3-O-(2,6-dirhamnosyl)-galactoside more effectively than red light. Short-term red light irradiation (12 and 36 h) significantly increased the levels of malonyl daidzin and malonyl genistin, the predominant isoflavones in soybeans, along with higher expressions of flavonoid biosynthetic genes. The accumulation of specialized metabolites under stress conditions is regulated at the molecular level by various genes and transcription factors, including phytohormonal pathways. Xu et al. found that treatment of *Tanacetum cinerariifolium* with phytohormones (MeJA, abscisic acid) significantly increased the expression of *TcbZIP60*, which was further shown to regulate both terpenoid and JA pathways of pyrethroid biosynthesis in *Tanacetum cinerariifolium*. Similarly, Xu et al. found that the content of aspalathoside VI in *Dipsacus asperoides* could be increased by MeJA treatment, and the results of transcriptome analysis showed that MeJA treatment could promote the expression of genes such as *acetyl-CoA acetyltransferase* (*DaAACT*), *3-hydroxy-3-methylglutaryl-coenzyme A synthase* (*DaHMGCS*), and 3*-hydroxy-3-methylglutaryl-coenzyme A reductase* (*DaHMGCR*) in triterpene biosynthesis pathway, and consequently promote the biosynthesis of aspalathoside VI.

In addition to the metabolism study, Zhang et al. verified the involvement of *CsPUPs* in the regulation of caffeine transport in tea trees by applying a heterologous expression system from yeast and Arabidopsis. On the other hand, obtaining high yields of valuable metabolites through synthetic biology is a goal we all aspire to. Nguyen et al. achieved efficient production of spirooxindoles by expressing a P450 enzyme from *Mitragyna speciosa* in *Saccharomyces cerevisiae*. This accomplishment paves the way for obtaining the well-known spectrum of spirooxindoles and their derivatives.

The articles in this Research Topic provide valuable insights into the biosynthesis and regulation of plant-specialized metabolites. There is a diversity of natural plant-specific metabolites, and the regulation mechanisms of their synthesis are unique and complicated. In the future, more studies should focus on the function of key genes of specific metabolite anabolism *in vitro* and *in vivo*, and reveal the synthesis mechanism and metabolic pathway of specific metabolites under different physiological environmental conditions.

## Author contributions

WZ: Writing – original draft. FX: Writing – review & editing. MK: Writing – review & editing.

